# ANKRD54 preferentially selects Bruton’s Tyrosine Kinase (BTK) from a Human Src-Homology 3 (SH3) domain library

**DOI:** 10.1371/journal.pone.0174909

**Published:** 2017-04-03

**Authors:** Manuela O. Gustafsson, Dara K. Mohammad, Erkko Ylösmäki, Hyunseok Choi, Subhash Shrestha, Qing Wang, Beston F. Nore, Kalle Saksela, C. I. Edvard Smith

**Affiliations:** 1 Department of Laboratory Medicine, Clinical Research Center, Karolinska Institutet, Karolinska University Hospital Huddinge, SE Stockholm, Sweden; 2 Department of Biology, College of Science, Salahaddin University-Erbil, Erbil, Kurdistan Region-Iraq; 3 Department of Virology, University of Helsinki and Helsinki University Hospital, Helsinki, Finland; 4 Department of Biochemistry, School of Medicine, Faculty of Medical Sciences, University of Sulaimani, Sulaimani, Iraq; 5 Department of Health, Kurdistan Institution for Strategic Studies and Scientific Research (KISSSR), Sulaimani, Kurdistan-Iraq; Universita degli Studi di Roma Tor Vergata, ITALY

## Abstract

Bruton’s Tyrosine Kinase (BTK) is a cytoplasmic protein tyrosine kinase with a fundamental role in B-lymphocyte development and activation. The nucleocytoplasmic shuttling of BTK is specifically modulated by the Ankyrin Repeat Domain 54 (ANKRD54) protein and the interaction is known to be exclusively SH3-dependent. To identify the spectrum of the ANKRD54 SH3-interactome, we applied phage-display screening of a library containing all the 296 human SH3 domains. The BTK-SH3 domain was the prime interactor. Quantitative western blotting analysis demonstrated the accuracy of the screening procedure. Revealing the spectrum and specificity of ANKRD54-interactome is a critical step toward functional analysis in cells and tissues.

## Introduction

Protein-protein interactions are prerequisite events for most cellular functions, since they organize signal transductions networks that control many aspects of cell biology [1,2]. While much on how cellular receptors trigger an intracellular response, through organizing signaling networks, is known, most of these domain-protein interaction events lack detailed spatial and temporal context [2,3].

Ankyrin repeat domains are one of the most abundant solenoid folds, each domain consisting of ≈33 amino acid stretches composed of a β-hairpin followed by two antiparallel α-helices and a variable loop [4]. Ankyrin repeat domains have been found in a plethora of proteins with a wide range of cellular functions involved in protein transport, cell-cell signaling, cytoskeleton integrity, cell-cycle regulation or development [5]. Despite sequence similarities in the ankyrin repeat domain, they are interacting with distinctive unrelated proteins by different mechanisms allowing them to function and locate in different intra- and/or extracellular locations [4,6], like GABPβ-GABPα-DNA complex in the nucleus [4], NF-κB/IκB system in the cytoplasm [7] and the intracellular domain of Notch receptor/cytosolic effector Deltex at the plasma membrane [8]. A unifying trait of the ankyrin repeat proteins is that they lack enzymatic activity and typically function as universal scaffolds or adaptor molecules.

Ankyrin Repeat Domain 54 (ANKRD54) consists of 4 ankyrin repeat domains, flanked by a nuclear localization signal (NLS) and a nuclear export signal (NES), and expressed in various tissues [9,10]. The secondary structure composition and measurements in the energetic signature of individual ankyrin repeats, reveals the GxTPLHLA motif as highly conserved within the internal and C-terminal repeats, whereas this motif is absent in the N-terminal repeats. The majority of the proteins contain 2 to 6 ankyrin repeats, while others can contain up to 29 [11].

Previously, we have reported ANKRD54 as a novel partner to BTK and shown that nuclear-resident BTK is specifically expelled by ANKRD54. Although, the precise role of BTK in the nucleus remains elusive, we have demonstrated that this mechanisms is mediated by the SH3 domain of BTK [9].

BTK is a non-receptor tyrosine kinase (nRTK) belonging to the TEC family of kinases together with other mammalian members, TEC, ITK, BMX/ETK and TXK/RLK. They play a pivotal role in regulating a wide range of cellular signaling pathways [12,13]. In humans, BTK is expressed in all hematopoietic cells except T-lymphocytes and plasma cells [14]. The BTK protein has a conserved multi-domain architecture comprised of Pleckstrin homology (PH) domain, Tec homology (TH) domain, Src homology 3 (SH3), Src homology 2 (SH2) and Kinase (SH1) domain in tandem. The BTK domains have the capacity to interact with cognate protein domains, polypeptide motifs, phosphoinositides and phosphorylated tyrosines, enabling BTK to carry out diverse multitasking biological processes. Two phosphorylated tyrosine residues, which are involved in these processes, have been identified in BTK, tyrosine-551 and tyrosine-223 within the kinase and SH3 domains, respectively [15–17].

Since the discovery of BTK mutations as the cause of X-linked agammaglobulinemia (XLA) [18,19], the role of BTK in B-cell signaling has been extensively studied [1–3,12,20–22]. Although, a huge number of protein partners have been reported to interact with various domains of BTK, only some of them were validated both *in vitro* and *in vivo*, while others have not been explored further [13,23].

SH3 domains are small interaction modules of approx. 60 amino acids found in many signaling proteins, including nRTKs. Evolutionarily they are well conserved mediating protein-protein interactions important for a variety of signal transduction processes [24]. It was determined that the human genome contain 296 different SRC homology-3 (SH3) domains [25]. The three-dimensional conformations of a number of SH3 domains [26,27] are well described and classified [28–30], predicting different modes of binding. Numerous low- and high- affinity binding partners have been characterized and cataloged [25,31–34].

Currently, a multiplicity of techniques such as, synthetic peptide-array screening, large-scale yeast two-hybrid screening or affinity selection of phage-display runs, together with computational approaches enable to characterize and compare the specificity of the “SH3 domain interactome” (i.e., the interaction of the SH3 domain with proteins) [33,34].

In this study, we explored a screening strategy based on phage-display libraries of the complete human SH3 domainome with ANKRD54, with the aim to identify the specificity of ANKRD54 for different SH3 domains.

## Materials and methods

### Plasmids

The ANKRD54 wild-type gene was cloned and prepared with extensions in order to generate compatible ends with the plasmid. For PCR amplification, the following primers were used: 5´- ATA TTT TCT AGA ATG GCA GCC GCC GCC GGG GAC; antisense 3’- TAC GTC TCG TAC CTC TTC TCC CCA TGG TCA GC, then digested with BglII and KpnI. To obtain the ANKRD54-Δ3 deletion mutant lacking the entire ankyrin repeat domain 3 (L176—A205), a site-directed deletion was generated, using a multiple overlapping-PCR strategy. The pEBB-vector expresses a biotinylation target-domain (BTD) fusion, defined by a single lysine residue that serves as the biotin acceptor. The BTD exists as biotin acceptor domain of the 1.3S subunit of *Propionibacterium shermanii* originating from the PinPoint (pp) Xa-1 vector, as described in [35]. The ANKRD54 wild-type and ANKRD54-Δ3 sequences were sub-cloned into the C-terminal position of the pEBB backbone (pEBB-pp-ANKRD54), which was cleaved with BamHI and KpnI.

The single BTK (P265A, Y268A) or double BTK (P265A/Y268A) mutants were created by single substitution mutagenesis and all plasmids were verified by sequencing (Mutagenex, Inc.). The cDNAs encoding wild-type BTK and nucleus-targeted GFP-BTK fusion protein containing a synthetic NLS signal located at C-terminus were generated by sub-cloning into pSGT or pEGFP vectors, as described earlier [9].

### Human SH3 domain phage display-based screening and protein production

SH3 phage libraries were prepared as described in [25] and the bio-panning procedure to select human SH3-domains binding to ANKRD54 was carried out according to [25] and [35]. Briefly, the biotinylated fusion protein of interest, ANKRD54 wild-type was analyzed with the ANKRD54-Δ3 mutant as a negative control and human immunodeficiency virus-1 (HIV Nef) as positive control, following transient transfection into HEK 293T cells. Cells were lysed and proteins were immobilized on M-280 streptavidin beads (Life Technologies), followed by the incubation with the infectious recombinant phages, already containing the SH3-phagemid vectors. After incubation and proper washing, the retained phages were eluted from the beads, followed by infection of *Escherichia coli* TG1 cells, and then plated at tenfold dilution. The identities of the SH3 domains of the selected phages were determined after sequencing of the clones.

The infectious recombinant phage supernatants were produced after overnight culture of *E*. *coli* cells carrying the phagemid(s) of interest, followed by super-infection with M13KO7 helper phage. After overnight growth of the double (ampicillin/kanamycin) resistant bacteria, the supernatants were cleared by filtration before usage.

### Cell culture and transfections

Namalwa (human Burkitt B-cell lymphoma), Cos7 (African green monkey kidney), HEK293T (human embryonal kidney) cell lines were obtained from the American Type Culture Collection (ATCC) and cultivated as previously described [9].

Transient transfections of Cos7 cells were performed using polyethylenimine (PEI) 25K (Polyscience, Inc) according to the manufacturer’s instructions and HEK293T cells were transfected with the standard calcium-phosphate precipitation method. The hematopoietic cells were transfected by electroporation using a Neon Electroporator (Life Technologies) and the 100μL tip kit with the following settings: 1350V (pulse-voltage); 20ms (pulse-width); 2 (pulse-number). Single cell suspensions were prepared from spleens obtained from normal and Btk knock-out (KO) mice. The use of mouse models in this study was approved by Stockholm South Animal Ethics Committee with registration number S56-14.

### Biotin-streptavidin pull-down assay and immunoprecipitation

All the pull-down assays regarding the ANKRD54-wt-BTD and the Δ3 mutant were conducted using Dynabeads MyOne Streptavidin-T1 (Life Technologies) together with DynaMag-2, Magnetic Particle-Concentrator, according to the manufacturer’s instructions. Briefly, cells were lysed and proteins were separated on gradient 4–12% SDS Bis-Tris NuPage gels and transferred onto nitrocellulose membranes using the iBlot system (Life Technologies). The membranes were then blocked with LI-COR Blocking Buffer and probed with specific primary antibodies: anti-Liar/ANKRD54 (ProSci Inc.), anti-BTK (BD Transduction Lab.), anti-Lyn44 (Santa Cruz) and IRDye-680 Streptavidin, and secondary antibodies: goat-anti rabbit 680LT/800CW and goat-anti mouse 680LT/800CW from LI-COR Biosciences. Western blots signals were scanned by using the Odyssey-Imager (LI-COR Biosciences).

### Immunofluorescence and microscopy

Immunofluorescence was performed as previously described [9]. Transfected cells were analyzed in a motorized Olympus IX-81 inverted-fluorescence microscope equipped with an XM10-monochrome-camera and narrow band-filter cubes for UV (DAPI), green (GFP) and red (Cy3) excitation. The cells were stained with a Cy3-conjugted goat anti-mouse IgG or fluorescein isothiocyanate (FITC)-conjugated anti-mouse IgG and nuclei were stained with DAPI (4’6-diamidino-2-phenylindole dihydrochloride).

### Statistical analysis

Data are reported as (M±SEM) and statistical analysis was carried out using SPSS version20 (SPSS Inc.). Comparisons between groups were assessed using one-way analysis of variance (ANOVA) in combination with Dunnett t-test *post hoc* analysis. P≤ 0.05 was considered statistically significant.

## Results

### Validation of biotinylation target-domain on ANKRD54 wt and ANKRD54-Δ3 mutant

**Fig 1:**
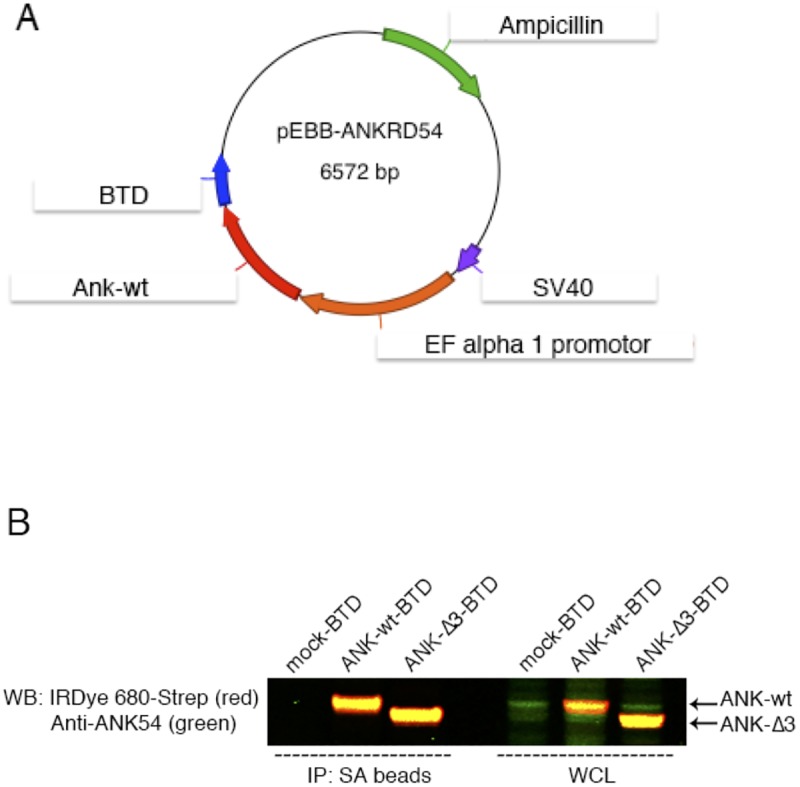
Strategy for protein-analysis and vector validation (A) Schematic representation of the pEBB vector containing biotinylation target-domain (BTD) and (B) Simultaneous two-color target analysis of ANKRD54 wt and Δ3 mutant in Cos7 cells. The first three lanes (from left side of the blot) represent immunoprecipitation (IP) with streptavidin (SA) beads and the last three lanes show whole cell lysate (WCL). Anti-ANKRD54 (green) primary antibody recognizes the C-terminus of the protein and anti-IRDye 680-Streptavidin (red) recognizes the BTD domain.

Validation of the newly generated vectors expressing biotinylation target-domain (BTD) on wild-type ANKRD54 (Fig 1A) and a mutant lacking the third ankyrin domain confirms that wild-type ANKRD54 protein specifically interacts with full length BTK. In order to biochemically verify these observations, we first transiently transfected the ANKRD54 wt or ANKRD54-Δ3 mutant into Cos7 cells, followed by immunoprecipitation with magnetic streptavidin-beads (SA) and immunoblotting. Anti-ANKRD54 antibody and IRDye-Streptavidin were used to visualize the proteins in two ways (Fig 1B). The pseudo-color-image (yellow indicates overlap of red and green) represents simultaneous detection of endogenous and exogenous ANKRD54 protein with biotinylation target-domain (ANKRD54-BTD) and demonstrates that the constructs are reliable.

**Fig 2:**
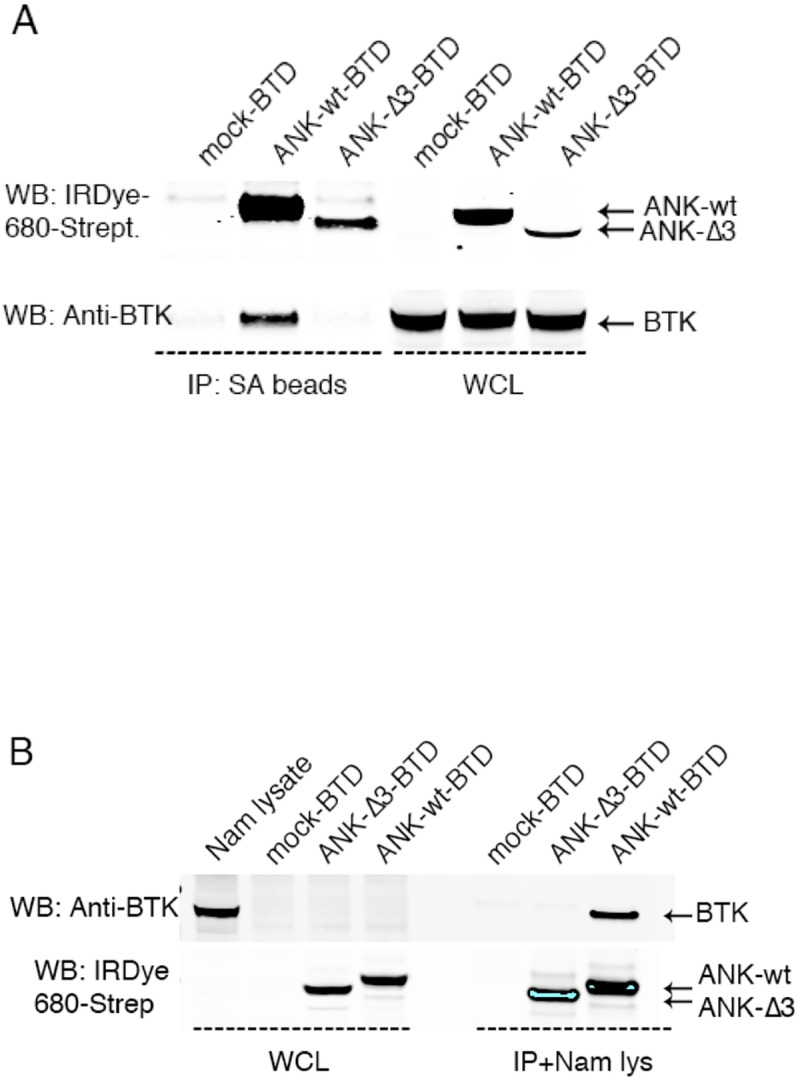
Characterization of the high-affinity ANKRD54/BTK interaction. (A) Transfection by electroporation of Namalwa cells and (B) transiently-transfected Cos7 cell lysate mixed with Namalwa (Nam) lysate, followed by co-immunoprecipitation (IP) of endogenous BTK. The missing expression of BTK from the whole cell lysate (WCL) of untransfected Cos7 cells (lane 1) shows that these cells do not express BTK.

Next, we set out to test whether functional expression of the ANKRD54-BTD wt and mutant constructs in B cells is feasible. Electroporation of Namalwa B-cells with the constructs resulted in the generation of stable proteins permitting successful immunoprecipitation, followed by pull-down of endogenous BTK using streptavidin-beads (Fig 2A). The ANKRD54-Δ3 mutant did not pull down endogenous BTK at all. Therefore, we used the ANKRD54-Δ3 mutant as negative control in all our experiments, including the phage-display screening.

Because the Cos7 cells do not express any BTK protein, as Namalwa B-cells do, we wanted to see if ANKRD54-BTD wt is able to specifically recognize and interact with endogenous BTK. Therefore, we extended our *in vitro* results and incubated the streptavidin-biotin complexes obtained after transient transfection of ANKRD54 wt and the Δ3 mutant in Cos7 cells with Namalwa B-cell lysate (Fig 2B). As expected, ANKRD54 wt was found to associate with BTK, while the ANKRD54-Δ3 mutant failed. Collectively these data clearly show that BTK and ANKRD54 interact in steady state, *in vitro*.

### *In vivo* characterization of BTK and ANKRD54 interactions

**Fig 3:**
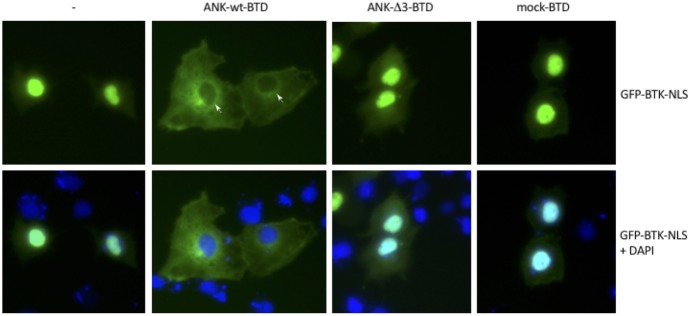
Nuclear exclusion of BTK-NLS fusion protein by ANKRD54. Subcellular localization of pEGFP-BTK-NLS alone (from left to right) and in the presence of wild-type ANKRD54, Δ3-mutant and mock. The white arrowheads in the second upper panel indicate nuclei devoid of BTK-NLS and the lower panels indicate merged images.

Furthermore, to prove that BTK can also interact with ANKRD54 in living cells, in terms of subcellular localization, we conducted immunofluorescence analysis with GFP-BTK-NLS. As previously reported by our group [9] this form of BTK is 100% localized in the nucleus of resting cells (Fig 3, left panel). Indeed, in the presence of the wild-type ANKRD54 a complete nuclear exclusion and cytoplasmic retention of BTK protein is observed (Fig 3 second panel, from left to right). Conversely, co-expression of BTK with ANKRD54-Δ3 mutant or empty-vector had no effect on the nuclear/cytoplasmic distribution of BTK. Thus, deletion of ankyrin domain 3 is sufficient to abolish the interaction with BTK. Consequently, these observations are in line with our previous findings.

### Phage-display screening for ANKRD54-binding SH3-domains

We next applied a phage-display screening using ANKRD54 as a bait against a complete human SH3-domain collection, according to the procedure described in [25]. Thus, we screened a library containing 296 different SH3-domains, derived from 217 human proteins. The excess of domains is due to the fact that several proteins contain more than one SH3-domain, namely up to 6. Sequences of all SH3-domains together with links to relevant bioinformatics information are available online [25].

After three screening rounds, 15 different SH3-domains were identified in total as ANKRD54 binders, but only two of them were previously described in the literature. These two SH3-domains belong to two kinases, namely BTK and LYN. Previously, Samuels [10] and colleagues have reported Liar (ANKRD54) as a novel partner to LYN influencing the erythropoietin-induced differentiation. As mentioned, we recently identified ANKRD54 as being the first protein specifically affecting the nucleocytoplasmic shuttling of BTK [9].

Scoring of these three rounds of experiments were calculated from the number of colony-hits (Table 1) and demonstrates clearly that the BTK-SH3-domain is predominantly (16 out 35 colonies) selected from the library (P-value < 10^−5^).

**Table 1:** SH3-domains identified by bio-panning of recombinant ANKRD54 against a human SH3-domain library.

Human SH3 domain-containing proteins identified	Number of hits (n = 35) from 3 experiments		
	Exp. 1	Exp. 2	Exp.3
**BTK–**Bruton’s tyrosine kinase, Tec family tyrosine kinase	5	5	6
**SNX33 –**Sorting nexin 33	0	0	3
**LYN**–LCK/YES novel tyrosine kinase, Src family tyrosine kinase	1	0	1
**PLCγ1** –Phospholipase C gamma 1	2	0	0
**BLK**–B-lymphoid tyrosine kinase, Src family tyrosine kinase	0	1	1
**TEC**–Tec protein tyrosine kinase	0	0	1
**SRC**–Sarcoma protein tyrosine kinase	0	1	0
**PTK6** –Protein tyrosine kinase 6	0	1	0
**NCF1** –Neutrophil cytosolic factor 1	1	0	0
**DLG3** –Discs, large homolog 3	0	0	1
**SH3YL1** –SH3 and SYLF domain containing 1	0	0	1
**ARHGAP26** –Rho GTPase activating protein 26	0	1	0
**GAS7** –Growth arrest specific 7	0	1	0
**BZRAP1** –Benzodiazepine receptor associated protein 1	0	0	1
**SH3KBP1 –**SH3 domain-containing kinase-binding protein 1	0	0	1
**Total**	9	10	16

### Characterization of low-score ANKRD54 binders

**Fig 4:**
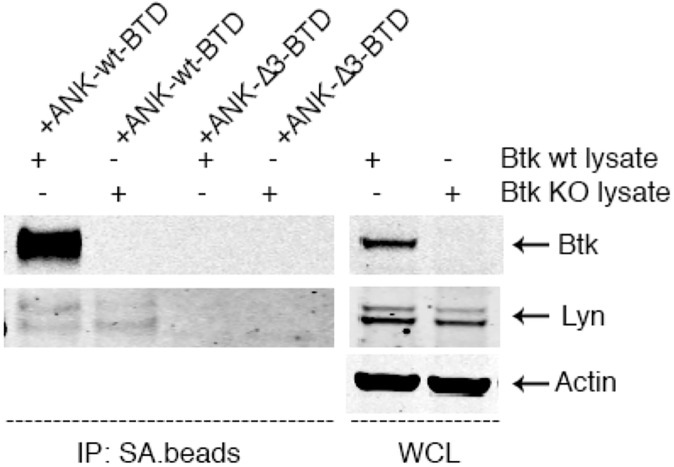
ANKRD54 selectively binds to BTK. Cos7 cells transiently-transfected with ANKRD54-wt or ANKRD54-Δ3 mutants followed by immunoprecipitation with streptavidin magnetic-beads. The IP complexes were incubated with whole cell lysates obtained from wt and Btk-KO primary spleen cells. The absence of BTK (second lane from left to right) did not enhance the enrichment to LYN. β-Actin used as quantity-index of protein, indicating equal-loading control of WCL.

In the next step, we wanted to biochemically examine the interaction between ANKRD54 and endogenously expressed proteins identified as low-score binders. First, Cos7 cells were transfected with biotinylated ANKRD54-wt or ANKRD54-Δ3 mutant followed by enrichment with streptavidin-beads. Subsequently, these beads were isolated and incubated with cell lysate obtained either from Namalwa B-cells or primary splenic mouse cells to determine the interaction with natively expressed proteins. Western blot detection of LYN, TEC, SRC, PLCγ1 or BLK revealed, apart that from BTK, only LYN could be validated to interact with ANKRD54 (Fig 4; negative results for other proteins, not shown).

To further explore this outcome, we decided to examine the ANKRD54/LYN interaction in the presence or absence of BTK. To achieve that we used cell lysates from Btk-KO and wt mouse splenic cells (Fig 4). The cell lysates were incubated with biotinylated ANKRD54-wt or the Δ3 mutant. In this set of experiments, the ANKRD54-Δ3 mutant was used as a negative control, instead of an empty vector-(mock). The absence of BTK did not enhance the enrichment of LYN, suggesting that access to ANKRD54 was not a limiting factor, and with the overall ANKRD54/LYN interaction being considerably weaker than that of BTK.

### Mutation on the BTK-SH3 3_10_ helix structure modulates ANKRD54 binding

From our previous work [9] and fine mapping analysis we knew that ANKRD54 binds to the BTK-SH3-domain in a polyproline-independent manner and only 22-amino-acids originating from the C-terminus of the SH3-domain were sufficient for this interaction.

**Fig 5:**
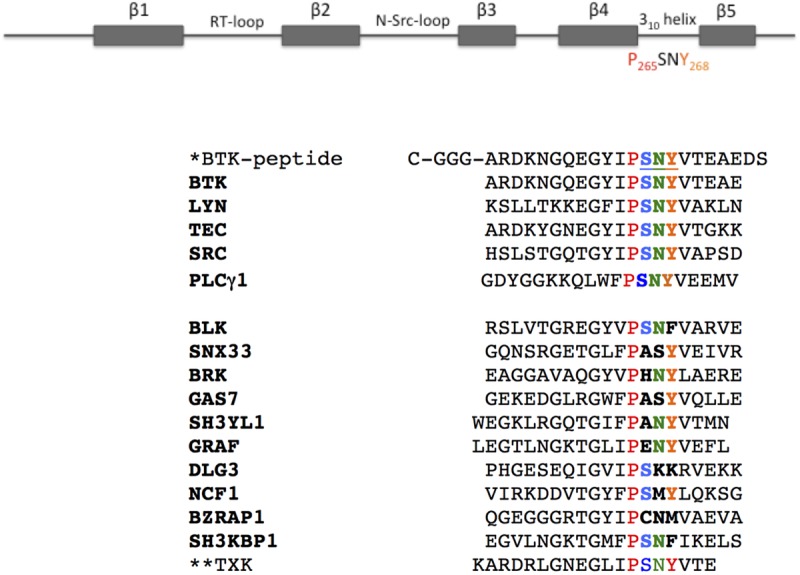
Schematic visualization of the SH3-domain and sequence-alignment of the C-terminal SH3-domains of the ANK54RD-binders identified in the screen. Secondary structural elements of BTK-SH3-domain and homology alignment of the last 20-amino-acids of the human SH3-domains, according to Kärkkäinen [25] database. The NCF1, BZRAP1 and SH3KBP1 proteins contain more than one SH3-domain and the sequence corresponds to the SH3-domain binding to ANKRD54. **SNY**-amino-acids create a right-handed 3_10_ helix [27]. *The biotinylated synthetic BTK-peptide (22-aa) reported in [9] was used as bait for the interaction with endogenous ANKRD54. **The TXK/RLK-SH3 was not identified in this screening, although ANKRD54 influenced the nucleocytoplasmic shuttling of full-length TXK [9].

In Fig 5, we present the amino-acid composition of the C-terminus of all the SH3-domains identified in the ANKRD54 binding screen, together with the synthetic BTK peptide used previously to pull-down endogenous ANKRD54 [9] and TXK. To study the effects of conserved residues on the BTK/ANKRD54 interaction, we generated single mutants for P265A, Y265A as well as the double mutant P265A/Y268A.

**Fig 6:**
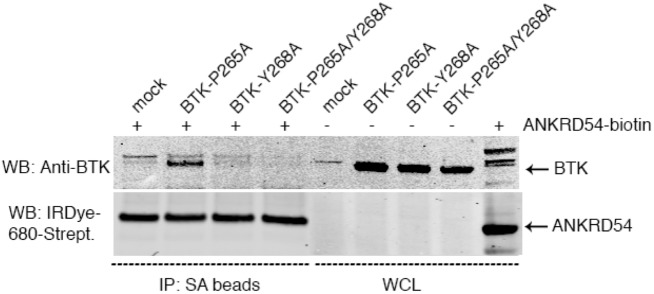
Residues in the 3_10_ helix important for the interaction of ANKRD54 to BTK. Cos7 cells were separately transfected with ANKRD54-wt and the following BTK-SH3 mutants: P265A, Y268A and P265A/Y268A. The obtained lysates were mixed and processed for immunoprecipitation.

ANKRD54-BTD-wt and BTK mutants were transiently transfected separately into Cos7 cells. After enrichment of ANKRD54 with streptavidin-beads, the immune-complexes were further incubated with each of the BTK mutant lysates. Immunoblotting analysis revealed that whenever the Y268 residue was mutated in BTK, the interaction with ANKRD54 was abolished (Fig 6). Substitution of the P265 residue had no effect on the interaction. The three-dimensional surface representation of amino-acids in the BTK-SH3-domain as determined by NMR shows that, the P265 residue is located in the inner cleft between the highly exposed W251 and Y268 residues [24].

## Discussion

Extensive studies have revealed that the binding specificity and cellular function of the plethora of SH3-domains are far more diverse than previously thought [27]. Except current canonical SH3-domain binding models, class I (R/K)xPxxP or class II PxxPx(R/K) motifs known to bind with relatively low-affinity ranging from 1μM to 1mM, there is Class III RxxK motifs proved to bind ligands with higher affinity [28]. Furthermore, the binding affinity to isolated singlets of SH3-domains compared with full-length, SH3-domain containing, intact proteins varies considerably [36]. The binding affinity of the full-length, intact, SH3-containing GADS protein to a complete proline-rich-region-peptide derived from SLP-76 was ≈ 10 fold-stronger than the interaction between only the C-terminal SH3 domain of GADS with the peptide derived from SLP-76 (≈ 250 nM) [27,29].

Previously, the classical mode of interaction to SH3 domains was thought to be via proline-rich-regions. Today, the thinking of the field is changing and an increased number of additional non-canonical binding modes between SH3-domains and peptides lacking PxxP motifs has been reported [27,36], including the interaction between endogenous ANKRD54 and a 22-amino-acids peptide derived from the C-terminal of the SH3-domain of BTK [9].

In this report, our objective was to examine the human SH3-domain interactome for ANKRD54, in order to identify additional SH3-domain-containing protein partners. Indeed, we provide strong evidence that ANKRD54 preferentially interacts with the BTK-SH3-domain. However, the structural basis for the ANKRD54 BTK-SH3-domain specificity and selectivity remains elusive. Additionally, to validate our phage-displayed SH3 domain interactome data to the targets, we performed a phage-ELISA binding assay. Although only relatively low level binding was observed when commercially available recombinant ANKRD54 adhered to plastic was used for this assay, the Btk-SH3 displaying phages did show the highest binding, and the signals obtained by six phages that were identified as ANKRD54 binders in library screening (BTK, LYN, SNX33, PLCγ1, BLK, and TEC; see Table 1) gave significantly (p = 0.02969 in a paired t-test) higher signals than two randomly chosen control SH3 phages (Hck and Eps8L1) across all phage dilutions tested (1:10, 1:20, 1:40, 1:80 and 1:160) (data not shown).

In our screenings, a number of new, much lower colony-score candidates for SH3-domain interactions was found: LYN, TEC, SRC, PLCγ1, BLK, SNX33, PTK6, NCF1, DLG3, SH3YL1, ARHGAP26, GAS7, BZRAP1 and SH3KBP1 (Table 1). We choose to give more attention to the proteins carrying the conserved PSNY-motif (BTK, LYN, TEC, SRC, PLCG1), in addition to BLK whose corresponding sequence is PSNF. In spite of the existence of a conserved 310helix motif, only BTK and LYN full-length proteins were confirmed to repeatedly interact with ANKRD54, in primary mouse spleen cells. Moreover, immunoprecipitation was also only positive for BTK and LYN using lysates of activated or resting isolated mouse B-cells or human Namalwa B-cells. This was furthermore confirmed in immunoprecipitates from exogenous interactions using proteins purified from co-transfected Cos7 cells.

We were also interested in exploring any possible competition between SH3-containg interactors, especially between LYN and BTK. We performed semi-quantitative IP in Btk-KO B-lymphocytes and did not find any increase in the LYN binding capacity in the absence of BTK. Except LYN, we could not confirm or validate any full-length ANKRD54 protein-interaction with low-score interactors: TEC, SRC, PLCγ1 or BLK. In this assay, we wanted to exclude the possibility that BTK outcompetes the LYN interaction, since the observed LYN band was weak. Indeed, the LYN interaction remained unaltered in the Btk-KO experiment, demonstrating that access to ANK54RD was not a limiting factor under our assay conditions; therefore, we conclude that the strong high-affinity interaction of ANKRD54 for BTK is remarkably selective.

However, for the other proteins identified in our triplicate screening procedure, we could not demonstrate any enhanced binding, suggesting that additional interactors with ANKRD54 are non-existing, at least from what we have tested (Data not shown). This was unexpected, since SH3-domain containing proteins often carry multiple domains as well as non-structured, adjacent regions flanking the SH3-domain permitting additional tethering. However, it is possible that other regions of a full-length protein mask the SH3-domain, thereby reducing binding to ANKRD54.

In an attempt to further explore the SH3-domain dependent interaction with the full-length ANKRD54, we generated a c-ABL mutant carrying the C-terminal portion of BTK’s SH3-domain by site-direct mutagenesis. Unpredictably, this mutant failed to interact with full-length ANKRD54. This observation suggests that the folding of other domain/motifs is required for intact interaction (Data not shown). This conclusion is supported by the report from Bartelt [28] and colleagues, where they proposed a revised model for the interaction of GRB2 with SH3-ligands, where a high affinity interaction occurs through other multiple contacts outside the proline-rich-region.

In addition to BTK and LYN, the binding of other possible ANKRD54 partners via their SH3-domains has been reported in the literature, such as TXK [9] or HS1, LASP1, VAV1, Hip55 and ESE2L [10], but they did not appear in our screening assay. We have previously shown that ANKRD54 influences the nucleocytoplasmic shuttling of full-length TXK, but in spite of that TXK displays the conserved PSNY-motif, this kinase was not identified in the SH3-interactome screen. This could be due to involvement of other interactions beyond the SH3-domain needed for stable interaction [28].

Additionally, mutation of highly conserved Y268 residue in the BTK-SH3-domain abolished the interaction with ANKRD54, indicating an important role of this residue for stable and selective interaction. In contrast, the availability of conserved P265 for protein-protein interaction may be limited and P265 is also located in a cleft. Thus, the preferential BTK selection in our library-screening relies on additional motif/domain interaction exists on BTK, which are not well-conserved or present on other low-score hits. Although our observation implies this notation, but further fine-tuning on multiple motif/module outside SH3 needed to be identified in future. Still, we can only speculate about the biological relevance of ANKRD54, but many questions remain to be answered in order to understand the basis for the ANKRD54 specificity toward BTK-SH3-domain.

In summary, these results extend our earlier understandings by showing that the interaction between BTK and ANKRD54 is highly selective, since it was also identified in a screen using human SH3-domainome. A novel finding is that BTK not only binds to ANKRD54, but stands out as the preferred interactor, being highly dominant over all other human SH3-domains.
